# The Role of MicroRNAs in Hyperlipidemia: From Pathogenesis to Therapeutical Application

**DOI:** 10.1155/2022/3101900

**Published:** 2022-06-17

**Authors:** Yu Xiang, Li Mao, Mei-Ling Zuo, Gui-Lin Song, Li-Ming Tan, Zhong-Bao Yang

**Affiliations:** ^1^The Affiliated Changsha Hospital of Hunan Normal University, Changsha, 410006 Hunan, China; ^2^Department of Basic Medicine, Changsha Health Vocational College, Changsha, 410600 Hunan, China; ^3^Institute of Emergency and Critical Care Medicine of Changsha, Changsha, China

## Abstract

Hyperlipidemia is a common metabolic disorder with high morbidity and mortality, which brings heavy burden on social. Understanding its pathogenesis and finding its potential therapeutic targets are the focus of current research in this field. In recent years, an increasing number of studies have proved that miRNAs play vital roles in regulating lipid metabolism and were considered as promising therapeutic targets for hyperlipidemia and related diseases. It is demonstrated that miR-191, miR-222, miR-224, miR-27a, miR-378a-3p, miR-140-5p, miR-483, and miR-520d-5p were closely associated with the pathogenesis of hyperlipidemia. In this review, we provide brief overviews about advances in miRNAs in hyperlipidemia and its potential clinical application value.

## 1. Introduction

Hyperlipidemia is a metabolic disorder with high morbidity and high mortality, usually characterized by lipid dysfunction and oxidative stress [1]. Hyperlipidemia is one of the recognized risk factors for cardiovascular disease (CVD) [2], where elevated low-density lipoprotein cholesterol (LDL-C) is considered a major factor in the development of atherosclerosis [3] and coronary heart disease [4]. It is estimated that the reduction of LDL-C by 10 mmol/L is associated with a 22% reduction in cardiovascular mortality and incidence rate, while the triacylglycerol (TG) concentration greater than 10 mmol/L is associated with a significant increase in risk of acute pancreatitis and cardiovascular disease [5, 6]. With the increasing use of miRNA microarrays and gene expression microarrays for hyperlipidemia gene expression profiling in recent years, we have gained a deeper understanding of the molecular biological mechanisms underlying the occurrence and development of hyperlipidemia. MicroRNAs are short (~21 nucleotides) noncoding RNA molecules that play an important role in the posttranscriptional regulation of gene expression in eukaryotes [7]. miRNAs are found in most eukaryotes [8] and commonly aberrantly expressed in human diseases [9]. Many studies have shown that miRNAs are involved in the regulation of a range of human diseases, including cancer, hepatitis, and cardiovascular diseases [10–12]. miRNAs have also been found involving in the pathogenesis of many allergic diseases, including asthma, eosinophilic esophagitis, allergic rhinitis, and eczema [13–15]. miRNAs are stable in the peripheral blood circulation and exhibit good physiological properties and can tolerate different temperatures, pH, storage times, and even repeated freezing and thawing [16]. It is well demonstrated that miRNAs play an important role in lipid metabolism and are important posttranscriptional regulators of genes that related with lipid homeostasis [17]. For example, previous studies identified miRNAs, such as miR-128 and miR-144, are regulators of plasma lipoprotein and cholesterol levels [18, 19]. Thus, discovery of specific hyperlipidemia-associated miRNAs may be a viable way to design miRNA-based therapies or obtain new prognostic markers in lipid metabolism-related diseases [20]. With the progressive research on the mechanism of small nucleic acids [21], it will greatly promote the translation of basic research into clinical practice and bring new opportunities for the development of drugs for the treatment of hyperlipidemia.

## 2. Hyperlipidemia

Hyperlipidemia is a disorder of lipid metabolism [22], usually with high levels of lipids in plasm because of the abnormal lipids transport and disturbed lipid metabolism [23]. Hyperlipidemia is characterized by elevated serum levels of total cholesterol (TC), triglycerides (TG), and low-density lipoprotein cholesterol (LDL-C) or reduced levels of high-density lipoprotein cholesterol (HDL-C) [24]. LDL-C is responsible for transporting fat molecules into cells, and if they are oxidized during transport, they can easily form plaques within the arterial walls, driving the progression of atherosclerosis. HDL-C helps the body to remove LDL from the arteries and brings it back to the liver to break it down, thus preventing cardiovascular disease [25, 26]. Hyperlipidemia is a serious health risk. Studies have shown that hyperlipidemia is involved in a range of diseases such as stroke, atherosclerosis, coronary heart disease, myocardial infarction, diabetes, and pancreatitis [22, 27–31] and is also closely associated with the development of neurodegenerative diseases such as Alzheimer's disease (AD) and Parkinson's disease (PD) [32]. Hyperlipidemia has a high prevalence in China, there were approximately 160 million patients with dyslipidemia in 2002, and this number is increasing [33]. In the United States, according to the 2011-2012 National Health and Nutrition Examination Survey, about 12.9% of adults over the age of 20 had excess total cholesterol [34]. More than 100 million people (about 53% of adults) have elevated LDL-C levels [35].

Hyperlipidemia can be divided into primary hyperlipidemia and secondary hyperlipidemia. Primary hyperlipidemia is characterized by a familial predisposition. Familial hypercholesterolemia (FH) is one of the most common monogenic dyslipidemias with a heterozygous prevalence of 1 : 250, which causes atherosclerosis and increases the risk of premature coronary artery disease (CAD) [36, 37]. An unhealthy diet and less physical activity are considered the most critical risk factors for hyperlipidemia [38]. Secondary hyperlipidemia is a dyslipidemia caused by other diseases, such as diabetes and hypertension. According to the Guidelines for the Prevention and Treatment of Dyslipidemia in Chinese Adults, when meeting one of the following criteria can be diagnosed as hyperlipidemia: TC ≥ 5.2 mmol/L, TG ≥ 1.7 mmol/L, LDL − C ≥ 3.4 mmol/L, non − HDL − C ≥ 4.1 mmol/L, or HDL − C < 1.0 mmol/L. Clinically, hyperlipidemia can be classified as hypercholesterolemia, hypertriglyceridemia, hyper-LDL-C, and mixed hyperlipidemia according to the difference of clinical indicators.

## 3. MicroRNAs

MicroRNAs (miRNAs) are endogenously transcribed noncoding RNAs, approximately 22 nt long, which are key regulators involving in many biological processes [39]. The first miRNA was identified in 1993 as a small RNA transcribed from the Caenorhabditis elegans lin-4 locus [40], and 7 years later, the first human miRNA let-7 was identified [41]. In humans, miRNAs involve in the expression of protein-coding genes and considered to be a complex gene expression modifier [42]. By binding to the 3′UTR of mRNA, miRNAs can posttranscriptionally inhibit mRNA translation into proteins or promote mRNA degradation [43, 44]. Based on such principles, scientists at the Sanger Institute established the microRNA Registry database to facilitate miRNAs research, later renamed miRbase in 2002 [45]. During the past decades, the number of discovered miRNAs increases year by year, and miRNAs have become a hot topic in medical research. Since miRNAs are negative regulator of genes, any change of the expression level of a certain miRNA may affect its corresponding target gene expression, even cellular homeostasis [46, 47]. Although many studies have shown changes in the expression levels of miRNAs in diseased states, their application as clinical biomarkers is still in its infancy [48, 49].

**Figure 1:**
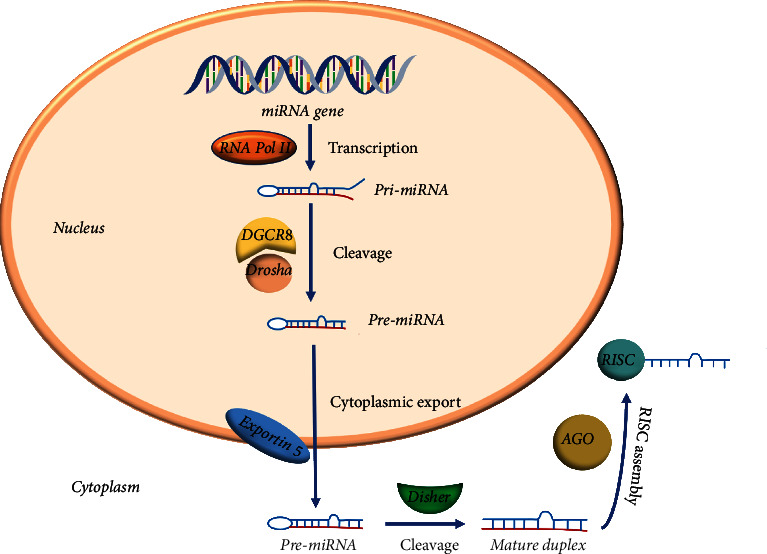
Overview of the miRNA biogenesis.

The biogenesis of miRNAs can be divided into multiple processes (Figure 1). First, synthesis of pri-miRNA is a large structure containing sequences of miRNAs and forms in the nucleus under the action of RNA polymerase II enzyme. The sequence of pri-miRNA may be an independent miRNA gene or parts of introns of protein coding for RNA polymerase II transcripts [50]. Then, the pri-miRNA transcript is cleaved in the nucleus by microprocessor, a catalytic complex composed of Drosha and Di George critical region 8 (DGCR8) [51, 52]. After that, the exportin 5 exports pre-miRNA to the cytoplasm where it is cleaved by Disher near the loop into small double-stranded RNAs [53]. This double-stranded RNA has a structure of 3′overhangs and strands that were named as guide strand and passenger strand according to their function, respectively, [54]. The guide strand, that is mature miRNA, was identified by AGO protein and forms the RNA-induced silencing complex (RISC) [55], while the passenger strand of the miRNA duplex is degraded [56, 57]. The RISC directs the miRNA to bind to its corresponding target mRNA and blocks gene expression by translational repression or mRNA degradation. This process is associated with the action of *a* about 8 nucleotides in length seed region of miRNA. The seed region will recognize the binding site of 3′UTR of mRNA by the way Watson-Crick complementary and lead to mRNA instability and finally translational repression [58, 59].

## 4. The Relationship between Hyperlipidemia and miRNA

It was demonstrated that miRNAs have important role in the development and progression of hyperlipidemia, which will open a new field for the study of hyperlipidemia [60]. Several miRNAs in patients with hyperlipidemia were found undergone altered, such as miRNA-191-3p, miRNA-933, miRNA-425-3p, and miR-208a [61]. This suggested that miRNAs may involves in the pathogenesis of hyperlipidemia. In vitro and in vivo studies have revealed that miRNAs play roles in controlling plasma LDL-C by regulating the expression of LDLR, such as miR-199a and miR-140 [62, 63]. Emerging evidence demonstrates that miRNAs involve in multiple processes of hypercholesterolemia, such as lipid synthesis (miR-122), fatty acid biosynthesis (miR-33), and lipoprotein formation and secretion (miR-27a) [64]. In addition, miRNAs involve in multiple processes of HDL metabolism, from synthesis to clearance [65]. Due to the key roles of miRNAs in lipid and lipoprotein metabolism, miRNAs have been considered as new therapeutic targets for lipid metabolism diseases [66, 67]. In fact, therapies based on miR-34 and mir-122 drugs are already in phase 2 clinical trial development [68, 69]. Thus, with the proliferation of human genomic and proteomic data and new delivery vectors development, miRNAs as therapeutic agents or therapeutic targets for hyperlipidemia will become a clinical reality.

### 4.1. miRNA Has Important Roles in the Pathogenesis of Hyperlipidemia

#### 4.1.1. miRNA and PCSK9

**Figure 2:**
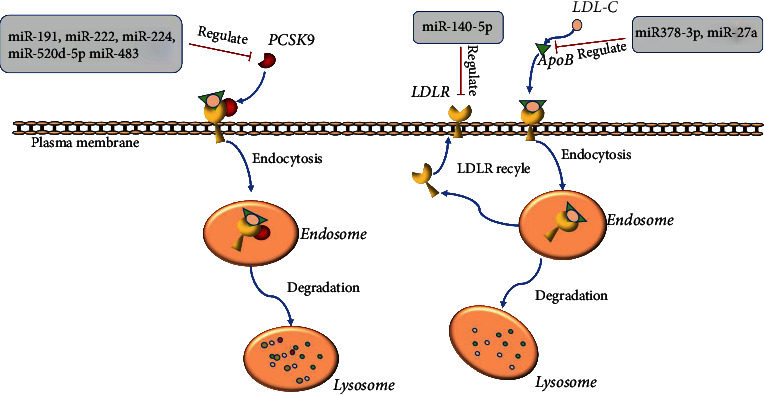
A schematic view of miR-mediated regulation of hyperlipidemia.

Proprotein convertase subtilisin/kexin type 9 (PCSK9) plays an important role in cholesterol metabolism by targeting LDLR [70, 71]. PCSK9 binds to LDLR on the cell surface to form the LDLR-PCSK9 complex, which hinders the endocytic recycling of LDLR for lysosomal degradation [72, 73]. This effectively reduces LDLR cell surface presentation and LDL-C endocytosis in hepatocytes, thereby increasing circulating LDL-C levels [74]. Previous studies have found that miRNAs involve in the process of formation of LDLR-PCSK9 complex, such as miR-191, miR-222, miR-224, miR-520d-5p, and miR-483, can suppress the expression of PCSK9 by directly binding to its 3′UTR and resulting in reduction of LDL-C levels [74–76] (Figure 2). Among them, miR-483 was one of the widely studied ones. In hyperlipidemic mice and humans, serum total cholesterol and LDL-C levels were found negatively correlated with miR-483-5p levels. The overexpression of miR-483 greatly reduced serum total cholesterol and LDL-C levels in mouse. In HepG2 cells [74], the high expression of miR-483-5p significantly inhibited the PCSK9 expression and led to LDLR upregulation and enhanced LDL-C uptake. In addition, it is reported that miR-337-3p could ameliorate the elevation of plasma LDL-C in mice feed with high-fat diet by inhibiting the expression of PCSK9 [77].

#### 4.1.2. miRNA and Sort1

In addition to the PCSK9 pathway, miRNAs, such as miR-122, miR-30c, and miR-140-5p, also have been reported to be negative regulator of some key factors in LDL-C metabolism and involve in the regulation of plasma LDL-C [78–80]. Sortilin 1 functions as an intracellular sorting receptor for apoB100 and was encoded by Sort1 gene [81]. Plasma ApoB100 levels are one of the strongest risk factors for coronary artery disease [81]. Previous studies have found that the increase of hepatic Sort1 can reduce hepatic apolipoprotein B (APOB) secretion and increase LDL catabolism, resulting in reduced plasma LDL-C and TG levels in mice [63, 82]. Emerging evidence has shown that miRNAs were involve in the expression regulation of ApoB100/Sort1 axis in animals. One of the miRNAs is miR378a-3p. In the study conducted by Zhang et al., they have demonstrated that the miR378a-3p expression is significantly increased in livers of hyperlipidemic mice. By targeted inhibit Sort1 expression, miR378a-3p can stabilize ApoB100 and promote its secretion, thereby facilitate VLDL secretion in the liver and exacerbate the pathogenesis of hyperlipidemia and hypolipoproteinemia [83]. Consistently, the overexpression of miR378a-3p increased the lipid droplet size and resulted in triglyceride accumulation in mice, while knockdown decreased triglyceride accumulation [84].

#### 4.1.3. miRNA and Cholesterol Metabolism

miRNAs also involve in cholesterol metabolism.

miR-33 was reported that it plays an important role in a variety of biological processes such as cholesterol homeostasis, HDL-cholesterol formation, and fatty acid oxidation [85]. miR-33a and miR-33b were significantly upregulated in the plasma of 28 hypercholesterolemia children compared to 25 healthy subjects, and both miRNAs were positively associated with the levels of TC and LDL-C. In the study by Price et al., they have demonstrated that inhibition of miR-33 increases HDL levels via promoting reverse cholesterol transport, and macrophage-specific loss of miR-33 decreases lipid accumulation and inflammation under hyperlipidemic conditions. This suggested that miR-33a is an important regulator of macrophage cholesterol efflux and HDL biogenesis and is a promising target for treatment of atherosclerosis [86, 87]. miR-33 also downregulates cholesterol efflux and HDL biogenesis by targeting ABC transport proteins (ABCA1: ATP-binding cassette subfamily A member 1, ABCG1: ATP-binding cassette subfamily B member 11) [88]. In a nonhuman primate model of dyslipidemia, miR-33 antagonism significantly reduced plasma VLDL-associated triglyceride levels, which was associated with the regulation of fatty acids (from synthesis to oxidation) [89].

miR-27 was demonstrated that it involves in hepatic lipid deposition, triglyceride synthesis, and lipoprotein uptake [90]. Studies have found that miR-27a can accelerate lipolysis by releasing more glycerol and free fatty acids from adipocytes and inhibit lipid storage in cells [91]. Accumulated evidences have shown that miR-27a inhibits the expression of many lipid metabolism genes, including fatty acid synthase (FASN), SREBP-1, SREBP-2, PPAR*α*, and PPAR*γ*, and ApoA1, ApoB100, and ApoE3 [92]. Therefore, miR-27a is an important regulator of lipid metabolism.

miR-128-3p has important roles in cholesterol efflux. In the study by Chandra et al., they have found that anti-miR-128-3p (AM-128) treatment inhibited the expression of miR-128-3p in hypercholesterolemic mouse and resulted in a significant reduction in circulating total cholesterol levels [93]. Consistently, in vitro studies [94] have demonstrated that miR-128-3p can promote cellular cholesterol accumulation by targeted inhibiting abca1, abcg1, and retinoid X receptor alpha (RXRA) and thereby inhibition of cholesterol efflux [94]. These data suggested that inhibition of microRNA-128-3p can attenuate hypercholesterolemia in animals.

In addition, there are evidences shown that miR-96/182/183 can targeted inhibit MED1/FBXW7 in hepatocytes [95, 96], and miR-122a antagonism [97] can inhibit cholesterol synthesis and reduce plasma cholesterol levels. This suggested that these miRNAs involve in lipid synthesis.

### 4.2. miRNA Involves in the Pathogenesis of Other Hyperlipidemia-Related Diseases

It is reported that miR-21a [98] is one of the first identified mammalian miRNAs involved in many physiological processes and multiple diseases, and one of its most representative roles is the regulation of lipid metabolism. Previous studies shown that the expression level of miR-21a-5p is downregulated in patients diagnosed as nonalcoholic fatty liver or in mice fed with high-fat diet, and knockdown of miR-21a-5p leads to hepatic steatosis, accelerated atherosclerosis, plaque necrosis, and vascular inflammation [99]. miR-200, miR-34a, miR-217, and miR-146a were reported to be highly expressed in endothelial cell senescence characterized by uncontrolled apoptosis, severe inflammation, and reduced endothelial nitric oxide synthesis and release, which was associated with endothelial dysfunction, atherosclerosis, and its complications [100]. miRNA-153 was upregulated in the pancreas of hypertriglyceridemia (HTG) animal models and in the plasma of HTG- Acute pancreatitis (AP) patients. The increase of miR-153 worsens AP and delays pancreatic repair in LPL dysfunction-induced HTG mice and its molecular mechanism associated with the inhibition of tumor necrosis factor receptor-associated factor 3 (TRAF3) [101]. Upregulation of miR-103 will inhibit endothelial cell proliferation, promote endothelial cell DNA damage, and consequently affect inflammatory response and promote atherosclerosis. MiR29c-3p is a negative regulator of dishevelled 2 (Dvl-2), a key mediator of the wnt/*β*-catenin signaling pathway. By inhibiting the expression of Dvl-2, miR29c-3p plays important roles in osteoblast differentiation in the hyperlipidemic setting. For example, the high expression of miR29c-3p causes implant osseointegration deficits [102, 103]. Similarly, by negatively regulating the expression of Mafb (vmaf myofascial fibrosarcoma oncogene family protein B), miR-155-5p improves *β*-cell adaptation to hyperlipidemic stress and compensates for obesity-induced insulin resistance and consequently limits the progression of obesity and atherosclerosis [104].

### 4.3. miRNAs Have Values in Hyperlipidemia Treatment

Although PCSK9 (proprotein convertase subtilisin/kexin type 9) inhibitors (monoclonal antibody) bring a new era on hyperlipidemia treatment, however, low safety profile and high cost limit its application [105–107]. Therefore, developing new drugs for hyperlipidemia is urgent for basic and clinical study. miRNAs are promising agents for their stability, controllability, good specificity, and operation simplicity. The recent studies shown that miRNAs are negative regulator of some key factors related to lipid metabolism or cholesterol metabolism, such as SREBP-1c (sterol regulatory element-binding transcription factor 1c), PPAR*α*, and NLRP3 (Table 1) [108–113]. Thus, miRNA as drugs or targets for hyperlipidemia therapy is theoretically possible. In fact, miRNAs as the action targets of drugs were studied broadly, such as grape seed proanthocyanidins, have an effect on the expression of miRNA-122 and miRNA-33 in rats, Averrhoa carambola free phenolic extract [114] has an effect on the expression level of miRNA-34a and miRNA-33 in db/db mice, paeonol (2′-hydroxy-4′-methoxyacetophenone, Pae) has an effect on the expression level of miRNA-223 in hyperlipidemic rats [115, 116], GNP (genipin) has an effect on the expression level of miR-142a-5p in rats [117], and diallyl trisulfide (DATS) has an effect on the miR-335 expression in obese rats [118]. In addition, even exercise has an effect on the expression of miR-21a-5p and exerts beneficial in hyperlipidemia [119, 120] [121]. Recently, therapies based on miR-34 and mir-122 drugs are already in phase 2 clinical trial development [68, 69]. These suggested that miRNAs attracted great attention by scientist and have potential values in hyperlipidemia treatment.

## 5. Summary and Outlook

The field of research on the use of miRNAs for hyperlipidemia is currently in its early stages. In most of the previous studies, the miRNA mimics were usually injected into target tissue sites and exert function. However, these methods cannot be used in clinic due to mimics easily be degraded by RNA enzymes in the blood. In addition, poor delivery of miRNA mimics to the target site makes it difficult to apply clinically. That is the reason why these studies of miRNAs successfully in animals cannot be translated into clinical applications [69, 123]. To overcome this above weakness of miRNAs, recently, scientists made some chemical modification of some nucleotide sequences of miRNAs to increase its stability and reduce its toxicity that it not essential for the intended function. As the advances in RNA chemical modification and delivery vector technologies have made continuously, several miRNAs have been entered into different clinical trials as therapeutic agents or therapeutic targets for human diseases. More and more studies have found that miRNAs play important roles in the pathogenesis of hyperlipidemia via regulating some key genes of lipid metabolism and are important biomarkers or target of hyperlipidemia. Thus, people paid great attention to using miRNAs for hyperlipidemia therapy. With the depth of miRNA research on hyperlipidemia and the advance of related technologies, such as delivery technology and materials science, we believe that it will be a success that miRNAs used for hyperlipidemia therapy clinically in the near future.

## Figures and Tables

**Table 1:** The relationship between miRNAs, targets, and disease.

miRNAs	Target	Disease or pathophysiological process	References
miR-122, miRNA-33, and miRNA-206 miR-142a-5p	SREBP-1c	Fatty acid metabolism	[64, 117, 122]
miR-34a	PPAR*α*	Hepatic steatosis	[109]
miR-223	NLRP3	Cholesterol metabolism	[116]
miR-128-3p	abca1, abcg1, and retinoid X receptor alpha (RXRA)	Hypercholesterolemia	[93, 94]
miR-27	(FASN), SREBP-1, SREBP-2, PPAR*α* and PPAR*γ*, and ApoA1, ApoB100, and ApoE3	Cholesterol metabolism	[92]
miR-33	ABCA1, ABCG1	Cholesterol metabolism	[88]
miR-337-3p, miR-191, miR-222, miR-224, miR-520d-5p, and miR-483	PCSK9	Lipid metabolism	[74–77]
miR-199a and miR-140	LDLR	Lipid metabolism	[64]

## Data Availability

The dataset used and/or analyzed during the current study are available from the corresponding author on reasonable request.

## References

[1] Nelson R. H. (2013). Hyperlipidemia as a risk factor for cardiovascular disease. *Primary Care*.

[2] Iqbal J., Al Qarni A., Hawwari A., Alghanem A. F., Ahmed G. (2018). Metabolic syndrome, dyslipidemia and regulation of lipoprotein metabolism. *Current Diabetes Reviews*.

[3] Hurtubise J., McLellan K., Durr K., Onasanya O., Nwabuko D., Ndisang J. F. (2016). The different facets of dyslipidemia and hypertension in atherosclerosis. *Current Atherosclerosis Reports*.

[4] Barrios V., Escobar C., Cicero A. F. (2017). A nutraceutical approach (Armolipid plus) to reduce total and LDL cholesterol in individuals with mild to moderate dyslipidemia: review of the clinical evidence. *Atherosclerosis. Supplements*.

[5] Harland J. I. (2012). Food combinations for cholesterol lowering. *Nutrition Research Reviews*.

[6] Nordestgaard B. G., Varbo A. (2014). Triglycerides and cardiovascular disease. *Lancet*.

[7] Paul S., Ruiz-Manriquez L. M., Ledesma-Pacheco S. J. (2021). Roles of microRNAs in chronic pediatric diseases and their use as potential biomarkers: a review. *Archives of Biochemistry and Biophysics*.

[8] Perron M. P., Provost P. (2008). Protein interactions and complexes in human microRNA biogenesis and function. *Frontiers in Bioscience: a Journal and Virtual Library*.

[9] Cortez M. A., Ivan C., Valdecanas D. (2016). PDL1 Regulation by p53 via miR-34. *JNCI: Journal of the National Cancer Institute*.

[10] Iorio M. V., Croce C. M. (2017). MicroRNAdysregulation in cancer: diagnostics, monitoring and therapeutics. A comprehensive review. *EMBO Molecular Medicine*.

[11] Baek J., Kang S., Min H. (2014). MicroRNA-targeting therapeutics for hepatitis C. *Archives of Pharmacal Research*.

[12] Gurha P. (2016). MicroRNAs in cardiovascular disease. *Current Opinion in Cardiology*.

[13] Weidner J., Bartel S., Kılıç A. (2021). Spotlight on microRNAs in allergy and asthma. *Allergy*.

[14] Dissanayake E., Inoue Y. (2016). MicroRNAs in allergic disease. *Current Allergy and Asthma Reports*.

[15] Zhang X. H., Zhang Y. N., Liu Z. (2014). MicroRNA in chronic rhinosinusitis and allergic rhinitis. *Current Allergy and Asthma Reports*.

[16] Xu J., Chen Z., Wang Y. (2019). Several circulating miRNAs related to hyperlipidemia and atherosclerotic cardiovascular diseases. *Lipids in Health and Disease*.

[17] Horie T., Baba O., Kuwabara Y. (2014). MicroRNAs and lipoprotein metabolism. *Journal of Atherosclerosis and Thrombosis*.

[18] Goedeke L., Wagschal A., Fernandez-Hernando C., Naar A. M. (2016). miRNA regulation of LDL-cholesterol metabolism. *Biochimica et Biophysica Acta (BBA)-Molecular and Cell Biology of Lipids*.

[19] Ramírez C. M., Rotllan N., Vlassov A. V. (2013). Control of cholesterol metabolism and plasma high-density lipoprotein levels by microRNA-144. *Circulation Research*.

[20] Barbalata T., Zhang L., Dulceanu M. D. (2020). Regulation of microRNAs in high-fat diet induced hyperlipidemic hamsters. *Scientific Reports*.

[21] Ogris M. (2010). Nucleic acid therapeutics: concepts for targeted delivery to solid tumors. *Therapeutic Delivery*.

[22] Mogensen S. S., Schmiegelow K., Grell K. (2017). Hyperlipidemia is a risk factor for osteonecrosis in children and young adults with acute lymphoblastic leukemia. *Haematologica*.

[23] Wang L., Zheng W., Yang J., Ali A., Qin H. (2022). Mechanism of Astragalus membranaceus alleviating acquired jyperlipidemia induced by high-fat diet through regulating lipid metabolism. *Nutrients*.

[24] Li P. C., Tsai I. J., Hsu C. Y. (2018). Risk of hyperlipidemia in women with hysterectomy-a retrospective cohort study in Taiwan. *Scientific Reports*.

[25] Gunness P., Gidley M. J. (2010). Mechanisms underlying the cholesterol-lowering properties of soluble dietary fibre polysaccharides. *Food & Function*.

[26] van der Gronde T., Hartog A., van Hees C., Pellikaan H., Pieters T. (2016). Systematic review of the mechanisms and evidence behind the hypocholesterolaemic effects of HPMC, pectin and chitosan in animal trials. *Food Chemistry*.

[27] Pawelczyk M., Chmielewski H., Kaczorowska B., Przybyla M., Baj Z. (2016). Platelet reactivity in patients with stroke and hyperlipidemia, GPIb*α* assessment. *Clinical and Applied Thrombosis/Hemostasis*.

[28] Yang Z. H., Pryor M., Noguchi A. (2019). Dietary palmitoleic acid attenuates atherosclerosis progression and hyperlipidemia in low-density lipoprotein receptor-deficient mice. *Molecular Nutrition & Food Research*.

[29] Guan W. J., Yang G. J. (2016). Significance of change of retinol binding protein 4 level of plasma of patients with coronary heart disease complicated with hyperlipidemia. *European Review for Medical and Pharmacological Sciences*.

[30] Yan L., Han P., Man J., Tian Y., Wang F., Wang J. (2021). Discovery of lipid profiles of type 2 diabetes associated with hyperlipidemia using untargeted UPLC Q-TOF/MS-based lipidomics approach. *Clinica Chimica Acta*.

[31] Zhu F., Guan Y., Zhang R. (2017). Inhibition of JAK2 signaling alleviates hyperlipidemia-intensified caerulin-induced acute pancreatitis in vivo. *Current Molecular Medicine*.

[32] Larsson S. C., Markus H. S. (2018). Does treating vascular risk factors prevent dementia and Alzheimer's disease? A systematic review and meta-analysis. *Journal of Alzheimer's Disease*.

[33] Du M. L., Wang Q. L., Zhao J., Zhou Y., Fang F. (2013). Investigation of serum lipid-related knowledge requirement and self-care behaviors of patients with hyperlipidemia. *Journal of Shanghai Jiaotong University*.

[34] Wang X., Seo Y. A., Park S. K. (2021). Serum selenium and non-alcoholic fatty liver disease (NAFLD) in US adults: National Health and Nutrition Examination Survey (NHANES) 2011–2016. *Environmental Research*.

[35] Liu Y., Liu F., Zhang L. (2021). Association between low density lipoprotein cholesterol and all-cause mortality: results from the NHANES. *Scientific Reports*.

[36] Vallejo-Vaz A. J., Kondapally Seshasai S. R., Cole D. (2015). Familial hypercholesterolaemia: a global call to arms. *Atherosclerosis*.

[37] Akioyamen L. E., Genest J., Shan S. D. (2017). Estimating the prevalence of heterozygous familial hypercholesterolaemia: a systematic review and meta-analysis. *BMJ Open*.

[38] Ma Y., Wang W., Zhang J. (2012). Hyperlipidemia and atherosclerotic lesion development in Ldlr-deficient mice on a long-term high-fat diet. *PLoS One*.

[39] Rahbar S., Pashaiasl M., Ezzati M. (2020). MicroRNA-based regulatory circuit involved in sperm infertility. *Andrologia*.

[40] Lee R. C., Feinbaum R. L., Ambros V. (1993). The C. elegans heterochronic gene lin-4 encodes small RNAs with antisense complementarity to lin-14. *Cell*.

[41] Pasquinelli A. E. (2013). The primary target oflet-7microRNA. *Biochemical Society Transactions*.

[42] Friedman R. C., Farh K. K. H., Burge C. B., Bartel D. P. (2009). Most mammalian mRNAs are conserved targets of microRNAs. *Genome Research*.

[43] Simionescu N., Niculescu L. S., Sanda G. M., Margina D., Sima A. V. (2014). Analysis of circulating microRNAs that are specifically increased in hyperlipidemic and/or hyperglycemic sera. *Molecular Biology Reports*.

[44] Lin X., Zhan J. K., Wang Y. J. (2016). Function, role, and clinical application of microRNAs in vascular aging. *BioMed Research International*.

[45] Zhong X., Heinicke F., Rayner S. (2019). miRBaseMiner, a tool for investigating miRBase content. *RNA Biology*.

[46] MacFarlane L. A., Murphy P. R. (2010). MicroRNA: biogenesis, function and role in cancer. *Current Genomics*.

[47] Carthew R. W., Sontheimer E. J. (2009). Origins and mechanisms of miRNAs and siRNAs. *Cell*.

[48] Bonneau E., Neveu B., Kostantin E., Tsongalis G. J., De Guire V. (2019). How close are miRNAs from clinical practice? A perspective on the diagnostic and therapeutic market. *Ejifcc*.

[49] Yaribeygi H., Atkin S. L., Sahebkar A. (2018). Potential roles of microRNAs in redox state: an update. *Journal of Cellular Biochemistry*.

[50] Çakmak H. A., Demir M. (2020). MicroRNA and cardiovascular diseases. *Balkan Medical Journal*.

[51] Gregory R. I., Yan K. P., Amuthan G. (2004). The microprocessor complex mediates the genesis of microRNAs. *Nature*.

[52] Lee Y., Ahn C., Han J. (2003). The nuclear RNase III Drosha initiates microRNA processing. *Nature*.

[53] Ha M., Kim V. N. (2014). Regulation of microRNA biogenesis. *Nature Reviews. Molecular Cell Biology*.

[54] Condrat C. E., Thompson D. C., Barbu M. G. (2020). miRNAs as biomarkers in disease: latest findings regarding their role in diagnosis and prognosis. *Cells*.

[55] Khan S., Ayub H., Khan T., Wahid F. (2019). MicroRNA biogenesis, gene silencing mechanisms and role in breast, ovarian and prostate cancer. *Biochimie*.

[56] Xie M., Steitz J. A. (2014). Versatile microRNA biogenesis in animals and their viruses. *RNA Biology*.

[57] Romero-Cordoba S. L., Salido-Guadarrama I., Rodriguez-Dorantes M., Hidalgo-Miranda A. (2014). miRNA biogenesis: biological impact in the development of cancer. *Cancer Biology & Therapy*.

[58] Lewis B. P., Shih I. H., Jones-Rhoades M. W., Bartel D. P., Burge C. B. (2003). Prediction of mammalian microRNA targets. *Cell*.

[59] Lai E. C. (2002). Micro RNAs are complementary to 3’ UTR sequence motifs that mediate negative post-transcriptional regulation. *Nature Genetics*.

[60] Zhou L., Irani S., Sirwi A., Hussain M. M. (2016). MicroRNAs regulating apolipoprotein B-containing lipoprotein production. *Biochimica et Biophysica Acta (BBA)-molecular and cell biology of lipids*.

[61] van Rooij E., Olson E. N. (2012). MicroRNA therapeutics for cardiovascular disease: opportunities and obstacles. *Nature Reviews. Drug Discovery*.

[62] Jamalzei B., Karami Tehrani F. S., Atri M. (2020). Evaluation of LDL receptor and scavenger receptor, class B, type 1 in the malignant and benign breast tumors: the correlation with the expression of miR-199a-5p, miR-199b-5p and miR-455-5p. *Gene*.

[63] Xu Y., Gao J., Gong Y. (2020). Hsa-miR-140-5p down-regulates LDL receptor and attenuates LDL-C uptake in human hepatocytes. *Atherosclerosis*.

[64] Yang Z., Cappello T., Wang L. (2015). Emerging role of microRNAs in lipid metabolism. *Acta Pharmaceutica Sinica B*.

[65] Canfrán-Duque A., Ramírez C. M., Goedeke L., Lin C. S., Fernández-Hernando C. (2014). microRNAs and HDL life cycle. *Cardiovascular Research*.

[66] Zhang T., Hu J., Wang X. (2019). MicroRNA-378 promotes hepatic inflammation and fibrosis via modulation of the NF-*κ*B-TNF*α* pathway. *Journal of Hepatology*.

[67] Musunuru K., Strong A., Frank-Kamenetsky M. (2010). From noncoding variant to phenotype via SORT1 at the 1p13 cholesterol locus. *Nature*.

[68] Qiu Z., Dai Y. (2014). Roadmap of miR-122-related clinical application from bench to bedside. *Expert Opinion on Investigational Drugs*.

[69] van Rooij E., Kauppinen S. (2014). Development of microRNA therapeutics is coming of age. *EMBO Molecular Medicine*.

[70] Yerushalmi B., Sokol R. J., Narkewicz M. R., Smith D., Ashmead J. W., Wenger D. A. (2004). NARC-1/PCSK9 and its natural mutants: zymogen cleavage and effects on the low density lipoprotein (LDL) receptor and LDL cholesterol. *Journal of Biological Chemistry*.

[71] Cohen J. C., Boerwinkle E., Mosley T. H., Hobbs H. H. (2006). Sequence variations in PCSK9, low LDL, and protection against coronary heart disease. *New England Journal of Medicine*.

[72] Lagace T. A., Curtis D. E., Garuti R. (2006). Secreted PCSK9 decreases the number of LDL receptors in hepatocytes and in livers of parabiotic mice. *The Journal of Clinical Investigation*.

[73] Zhang D. W., Lagace T. A., Garuti R. (2007). Binding of proprotein convertase subtilisin/kexin type 9 to epidermal growth factor-like repeat A of low density lipoprotein receptor decreases receptor recycling and increases degradation. *Journal of Biological Chemistry*.

[74] Dong J., He M., Li J. (2020). microRNA-483 ameliorates hypercholesterolemia by inhibiting PCSK9 production. *JCI Insight*.

[75] Naeli P., Mirzadeh Azad F., Malakootian M., Seidah N. G., Mowla S. J. (2017). Post-transcriptional regulation of PCSK9 by miR-191, miR-222, and miR-224. *Frontiers in Genetics*.

[76] Salerno A. G., van Solingen C., Scotti E. (2020). LDL receptor pathway regulation by miR-224 and miR-520d. *Front Cardiovasc Medicine*.

[77] Xu X., Dong Y., Ma N. (2021). MiR-337-3p lowers serum LDL-C level through targeting PCSK9 in hyperlipidemic mice. *Metabolism*.

[78] Arca M. (2019). PCSK9 inhibitors (PCSK9i), a new opportunity for cardiovascular prevention: clinical and regulatory aspects and access to therapy. *Recenti Progressi in Medicina*.

[79] Goedeke L., Aranda J. F., Fernández-Hernando C. (2014). MicroRNA regulation of lipoprotein metabolism. *Current Opinion in Lipidology*.

[80] Al-Rawaf H. A. (2019). Circulating microRNAs and adipokines as markers of metabolic syndrome in adolescents with obesity. *Clinical Nutrition*.

[81] Li J., Bi L., Hulke M., Li T. (2014). Fish oil and fenofibrate prevented phosphorylation-dependent hepatic sortilin 1 degradation in western diet-fed mice. *Journal of Biological Chemistry*.

[82] Strong A., Ding Q., Edmondson A. C. (2012). Hepatic sortilin regulates both apolipoprotein B secretion and LDL catabolism. *The Journal of Clinical Investigation*.

[83] Huang N., Wang J., Xie W. (2015). miR-378a-3p enhances adipogenesis by targeting mitogen-activated protein kinase 1. *Biochemical and Biophysical Research Communications*.

[84] Zhang T., Shi H., Liu N. (2020). Activation of microRNA-378a-3p biogenesis promotes hepatic secretion of VLDL and hyperlipidemia by modulating ApoB100-Sortilin1 axis. *Theranostics*.

[85] Martino F., Carlomosti F., Avitabile D. (2015). Circulating miR-33a and miR-33b are up-regulated in familial hypercholesterolaemia in paediatric age. *Clinical Science (London, England)*.

[86] Price N. L., Rotllan N., Canfrán-Duque A. (2017). Genetic dissection of the impact of miR-33a and miR-33b during the progression of atherosclerosis. *Cell Reports*.

[87] Rayner K. J., Sheedy F. J., Esau C. C. (2011). Antagonism of miR-33 in mice promotes reverse cholesterol transport and regression of atherosclerosis. *The Journal of Clinical Investigation*.

[88] Rayner K. J., Suárez Y., Dávalos A. (2010). miR-33 contributes to the regulation of cholesterol homeostasis. *Science*.

[89] Rayner K. J., Esau C. C., Hussain F. N. (2011). Inhibition of miR-33a/b in non-human primates raises plasma HDL and lowers VLDL triglycerides. *Nature*.

[90] Xie W., Li L., Zhang M. (2016). MicroRNA-27 prevents atherosclerosis by suppressing lipoprotein lipase-induced lipid accumulation and inflammatory response in apolipoprotein E knockout mice. *PLoS One*.

[91] Sedgeman L. R., Michell D. L., Vickers K. C. (2019). Integrative roles of microRNAs in lipid metabolism and dyslipidemia. *Current opinion in lipidology*.

[92] Yerlikaya F. H., Can U., Alpaydin M. S., Aribas A. (2019). The relationship between plasma microRNAs and serum trace elements levels in primary hyperlipidemia. *Bratislavské Lekárske Listy*.

[93] Chandra A., Sharma K., Pratap K., Singh V., Saini N. (2021). Inhibition of microRNA-128-3p attenuates hypercholesterolemia in mouse model. *Life Sciences*.

[94] Adlakha Y. K., Khanna S., Singh R., Singh V. P., Agrawal A., Saini N. (2013). Pro-apoptotic miRNA-128-2 modulates ABCA1, ABCG1 and RXR *α* expression and cholesterol homeostasis. *Cell Death & Disease*.

[95] Sedgeman L. R., Beysen C., Allen R. M., Ramirez Solano M. A., Turner S. M., Vickers K. C. (2018). Intestinal bile acid sequestration improves glucose control by stimulating hepatic miR-182-5p in type 2 diabetes. *American Journal of Physiology-Gastrointestinal and Liver Physiology*.

[96] Jeon T. I., Esquejo R. M., Roqueta-Rivera M. (2013). An SREBP-responsive microRNA operon contributes to a regulatory loop for intracellular lipid homeostasis. *Cell Metabolism*.

[97] Elmén J., Lindow M., Silahtaroglu A. (2008). Antagonism of microRNA-122 in mice by systemically administered LNA-antimiR leads to up-regulation of a large set of predicted target mRNAs in the liver. *Nucleic Acids Research*.

[98] Krichevsky A. M., Gabriely G. (2009). miR-21: a small multi-faceted RNA. *Journal of Cellular and Molecular Medicine*.

[99] Sun C., Huang F., Liu X. (2015). miR-21 regulates triglyceride and cholesterol metabolism in non-alcoholic fatty liver disease by targeting HMGCR. *International Journal of Molecular Medicine*.

[100] Arunachalam G., Upadhyay R., Ding H., Triggle C. R. (2015). MicroRNA signature and cardiovascular dysfunction. *Journal of Cardiovascular Pharmacology*.

[101] Dai J., Jiang M., Hu Y. (2021). Dysregulated SREBP1c/miR-153 signaling induced by hypertriglyceridemia worsens acute pancreatitis and delays tissue repair. *JCI Insight*.

[102] Lee Y. N., Gao Y., Wang H. Y. (2008). Differential mediation of the Wnt canonical pathway by mammalian dishevelleds-1, -2, and -3. *Cellular Signalling*.

[103] Huang X., Wang Z., Li D. (2018). Study of microRNAs targeted Dvl2 on the osteoblasts differentiation of rat BMSCs in hyperlipidemia environment. *Journal of Cellular Physiology*.

[104] Zhu M., Wei Y., Geißler C. (2017). Hyperlipidemia-induced microRNA-155-5p improves *β*-cell function by targetingMafb. *Diabetes*.

[105] Sabatine M. S., Giugliano R. P., Keech A. C. (2017). Evolocumab and clinical outcomes in patients with cardiovascular disease. *New England Journal of Medicine*.

[106] Waters D. D., Hsue P. Y. (2017). PCSK9 inhibition to reduce cardiovascular risk. *Circulation Research*.

[107] Burke A. C., Dron J. S., Hegele R. A., Huff M. W. (2017). PCSK9: regulation and target for drug development for dyslipidemia. *Annual Review of Pharmacology and Toxicology*.

[108] Cheung O., Puri P., Eicken C. (2008). Nonalcoholic steatohepatitis is associated with altered hepatic microRNA expression. *Hepatology*.

[109] Ding J., Li M., Wan X. (2015). Effect of miR-34a in regulating steatosis by targeting PPAR*α* expression in nonalcoholic fatty liver disease. *Scientific Reports*.

[110] Ferré P., Foufelle F. (2007). SREBP-1c transcription factor and lipid homeostasis: clinical perspective. *Hormone Research*.

[111] Lin J., Yang R., Tarr P. T. (2005). Hyperlipidemic effects of dietary saturated fats mediated through PGC-1*β* coactivation of SREBP. *Cell*.

[112] He Z., Hu C., Jia W. (2016). miRNAs in non-alcoholic fatty liver disease. *Frontiers in Medicine*.

[113] Wu H., Zhang T., Pan F. (2017). MicroRNA-206 prevents hepatosteatosis and hyperglycemia by facilitating insulin signaling and impairing lipogenesis. *Journal of Hepatology*.

[114] Pang D., You L., Zhou L., Li T., Zheng B., Liu R. H. (2017). Averrhoa carambola free phenolic extract ameliorates nonalcoholic hepatic steatosis by modulating mircoRNA-34a, mircoRNA-33 and AMPK pathways in leptin receptor-deficient db/db mice. *Food & Function*.

[115] Novák J., Olejníčková V., Tkáčová N., Santulli G. (2015). Mechanistic role of MicroRNAs in coupling lipid metabolism and atherosclerosis. *Advances in Experimental Medicine and Biology*.

[116] Shi X., Xie X., Sun Y. (2020). Paeonol inhibits NLRP3 mediated inflammation in rat endothelial cells by elevating hyperlipidemic rats plasma exosomal miRNA-223. *European Journal of Pharmacology*.

[117] Zhong H., Chen K., Feng M. (2018). Genipin alleviates high-fat diet-induced hyperlipidemia and hepatic lipid accumulation in mice via miR-142a-5p/SREBP-1c axis. *The FEBS Journal*.

[118] Miura A., Ikeda A., Abe M. (2021). Diallyl trisulfide prevents obesity and decreases miRNA-335 expression in adipose tissue in a diet-induced obesity rat model. *Molecular Nutrition & Food Research*.

[119] Soci U. P. R., Fernandes T., Hashimoto N. Y. (2011). MicroRNAs 29 are involved in the improvement of ventricular compliance promoted by aerobic exercise training in rats. *Physiological Genomics*.

[120] Xiao J., Bei Y., Liu J. (2016). miR-212 downregulation contributes to the protective effect of exercise against non-alcoholic fatty liver via targeting FGF-21. *Journal of Cellular and Molecular Medicine*.

[121] Zhao J., Song Y., Zeng Y. (2021). Improvement of hyperlipidemia by aerobic exercise in mice through a regulatory effect of miR-21a-5p on its target genes. *Scientific Reports*.

[122] Baselga-Escudero L., Bladé C., Ribas-Latre A. (2012). Grape seed proanthocyanidins repress the hepatic lipid regulators miR-33 and miR-122 in rats. *Molecular Nutrition & Food Research*.

[123] Li Z., Rana T. M. (2014). Therapeutic targeting of microRNAs: current status and future challenges. *Nature Reviews. Drug Discovery*.

